# Pharmacokinetics Study of Recombinant Hirudin in the Plasma of Rats Using Chromogenic Substrate, ELISA, and Radioisotope Assays

**DOI:** 10.1371/journal.pone.0064336

**Published:** 2013-06-13

**Authors:** Su-yun Jiang, Jian Jiao, Ting-ting Zhang, Yong-ping Xu

**Affiliations:** 1 Dalian University of Technology, Dalian, China; 2 Dalian Institute for Food and Drug Control, Dalian, China; University of North Dakota, United States of America

## Abstract

**Aim:**

To compare the analytical methods used to study the pharmacokinetics of recombinant hirudin in the plasma of rats that had been injected with ^125^I-recombinant hirudin.

**Methods:**

2.0 mg/kg ^125^I-recombinant hirudin were injected intravenously into rats. The recombinant hirudins in the plasma was analyzed by chromogenic substrate assay, enzyme-linked immunosorbent assay (ELISA), total radioisotope assay (RA) and trichloroacetic acid pre-treated total radioisotope assay (TCA-RA).

**Results:**

The chromogenic substrate assay standard curve was linear over the concentration range from 3.12 to 40.00 ng/ml for the recombinant hirudin in plasma. The relative standard deviations (RSD) for the intra- and inter-day variation were 5.0 to 6.3% and 11.9 to 12.6%, respectively. The recoveries of recombinant hirudin was 89.8% to 100.7%. The limit of quantification (LOQ) was 3.12 ng/ml. The concentration-time curve of the recombinant hirudin in the plasma could be explained as a two-compartment model. Pharmacokinetic parameters, including the half-life of distribution phase (t_1/2_ α), the half-life of elimination phase (t_1/2_ β), volume of apparent distribution (Vd), and area under the concentration-time curve from zero to infinite time (AUC_0–t_) were 7.59 min, 46.99 min, 0.17 L/kg, and 204.5 mg/L/min, respectively, as determined by chromogenic substrate assay; 6.41 min, 47.28 min, 1.24 L/kg, and 575.18 mg/L/min, respectively, as determined by ELISA; 3.69 min, 701.90 min, 0.04 L/kg, and 4189 mg/L/min, respectively as determined by RA; and 4.57 min, 724.9 min, 0.09 L/kg, and 2329 mg/L/min, respectively, as determined by TCA-RA.

**Conclusions:**

The chromogenic substrate assay on the concentration dynamics of the recombinant hirudin in the plasma is a specific, sensitive, and accurate analytical method for pharmacokinetic studies. Moreover, the pharmacokinetic parameters determined by the chromogenic substrate assay and ELISA are congruent except for AUC.

## Introduction

Hirudin is a small anti-coagulant protein originally isolated from the saliva of medicinal leeches [1]. Hirudin consists of 65 amino acid residues, with a molecular weight of about 7000 Da, and possesses anti-thrombin activity [2]. In contrast to heparin, hirudin does not require cofactors for thrombin inhibition [3]. Hirudin is the most powerful thrombin inhibitor with both anticoagulant and antithrombotic effects and is superior to the traditional anticoagulant heparin for treating thrombotic diseases [4]. A recombinant form of hirudin, in which the residue tyrosine 63 was replaced with a SO_3_-base form of the residue, exhibits similar pharmacological activity as that of the wildtype [5], [6].

The bioassay, immunoassay and widely used isotope tracer techniques [7], [8], [9] have been reported to be used in rH pharmacokinetic studies. The total radioactivity detected in the isotope tracer techniques does not represent the real hirudin concentration in blood samples and therefore other methods should be adopted for the pharmacokinetic study [9]. We have used enzyme-linked immunosorbent assay (ELISA) to study the pharmacokinetic parameters of recombinant hirudin [10]. Most recently, chromogenic substrate assay method was employed to study the metabolites of recombinant hirudin in rats [11]. However, the pharmacokinetic parameters determined from these assays vary, depending on the assay employed [9]. In Markwardt's study [12], the thrombin time detection and chromogenic substrate method were used to for rH pharmacokinetic research in dog and human plasma, and the elimination half-life (t_1/2β_) of the pharmacokinetic parameters calculated was 73 min for dog and 60–100 min for human. Richter et al [9] reported the t_1/2β_ was 65 min for rats and 56.6 min for dog by using the ^125^I-rH combined with thrombin titration assay. The t_1/2β_ of rH in baboons was determined as 24 min and 23 min by using the ^131^I-labeled rH combined the ecarin Clotting Time (ECT) method by Meiring et al [13]. Recently, Huang et al [8] reported that in rats, the t_1/2β_ was 172.8 min by the total plasma ^125^I-labeled rH radioactivity (RA) method and 107 min by the trichloroacetic acid precipitate plasma ^125^I-labeled rH radioactive activity (TCA-RA) method. Importantly, the pharmacokinetic parameter t_1/2β_ of rH varies not only between different experimental animals (73 min for dog; 60–100 min for human; 65 min for rat; 56.6 min for dog; 24 min for baboons; and 107–172 min for rats), but also in the same animals when different methods are employed (172 min by the RA vs. 102 min by the TCA-RA). When different methods are used for rH analysis in same animals, the pharmacokinetic parameters differences may be found by analyzing and comparing data determined by these methods. In the present study, we analyze the ^125^I-recombinant hirudin in the plasma of the rats that had been injected with the radiolabeled recombinant hirudinuse, using chromogenic substrate assay, ELISA, total radioisotope assay (RA), and trichloroacetic acid pre-treated total radioisotope assay (TCA-RA). By comparing the pharmacokinetics parameters obtained from these assays, we determine the optimal assay to study the pharmacokinetics of recombinant hirudin.

## Materials and Methods

### Ethics Statement

Animals were maintained and experiments were conducted in accordance with the Institutional Animal Care and Use Committee, Dalian University of Technology, and with the 1996 Guide for the Care and Use of Laboratory Animals (Institute of Laboratory Animal Resources on Life Sciences, National Research Council, National Academy of Sciences, Washington DC). The study was approved by the Education and Research Committee and the Ethics Committee of Dalian University of Technology (approval # DUT2010553A).

### Drugs and reagents

The recombinant hirudin used in this study (Lot Number 99110) was provided by the Dalian Gaoxin Biopharmaceutical Company (Dalian, China). The recombinant hirudin was dissolved in saline to 1.0 mg/ml. This standard stock solution was stored at −30°C.

^125^I-recombinant hirudin was radiolabeled and prepared using the modified chloraine-T method with a specific activity of 45.3±1.48 MBq/ml. The purity of the ^125^I-recombinant hirudin was determined to be 99.4±0.47% by HPLC (Chinese Academy of Military Medical Sciences; Beijing, China). The biological activity of the ^125^I-recombinant hirudin remained unchanged during the labeling procedure. Thrombin (1000 U/vial) was purchased from Sigma (St Louis, MO).

ELISA assays were conducted using the IMUBIND Hirudin Chromogenic Substrate Assay Kit (American Diagnostic Inc. Hauppauge, NY; Product #853). The kit included a rabbit anti-hirudin polyclonal antibody as the capture antibody, a horseradish peroxidase (HRP)-conjugated murine monoclonal antibody against hirudin, perborate/3, 3, 5, 5, -tetramethylbenzidine as the substrate, BPS buffer pH 7.4 with 0.05% Tween 20, and lyophilized hirudin standard. 0.5 M H_2_SO_4_ solution was self-prepared. Bovine serum albumin (BSA) was obtained from Sigma (St Louis, MO).

Chromozym chromogenic substrate (Tosyl-glycyl-polyl-arginine-4-nitrniline-acetate, TGPApNA), 20 mg/vial, sold under the brand name Chromozym TH, was purchased from Roche (Mannheim, Germany; Lot Number 70147021).

### Animals and Animal experiments

Male Sprague Dawley rats, weighing 190±10 g, were supplied by the Animal Center of the Chinese Academy of Military Medical Sciences (Beijing, China; SCXK (a) 2002–0001).

The rats were anesthetized with ether and cannulated in the carotid artery. After recovered from anesthesia, the rats were administered intravenously with 2.0 mg/kg of ^125^I-recombinant hirudin (0.4 g/L, 725 kBq/mL). A total of 0.5 mL of plasma was collected from the carotid artery intubation and added into centrifuge tubes pre-coated with 50 µl of 3.8% sodium citrate at 0-, 5-, 15-, 30-, 45-, 60-, 90-, 120-, 180-, 240- and 300-minute time points after the administration of recombinant hirudin. The plasma was clarified by centrifuging at 8000 rpm for 10 minutes. The supernatant was stored at −30°C.

### Instruments and Equipment

HPLC: LC-20AT, SPD-20A Detector (Shimadzu Company, Kyoto, Japan); Wizard 1470 Automatic (Perkin Elmer Company, Norwalk, CT); Microplate Reader EIx800, BIO-TEK Instruments (Winooski, VT); Size exclusion column, TSK-GEL G2000SW (TOSOH CORPORATION, Tokyo, Japan); C8 column, Kromasil B080806 (Elite Company, Dalian, China); Incubator, PYX-YDH-40×50 (Guang Medical Instrument Factory, Guangzhou, China); Centrifuges: TGL-16B (Tianjin Pharmaceutical Company, Tianjin, China); Gamma Counter, Wizard 1470 Automatic (Perkin Elmer Company, Waltham, MA).

### 1. Chromogenic Substrate Assay Method

#### Basic principle

This method is based on the principle that thrombin hydrolyzes the chromogenic substrate TGPApNA and releases pNA, which has an absorption at 405 nm. The thrombin is present in excess relative to the recombinant hirudin in the reaction. The quantity of recombinant hirudin is calculated from the difference between the total thrombin and the residual thrombin.

#### Preparation of solutions

Thrombin was diluted to 10 U/ml with 50 mM Tris-buffer pH 8.3 (containing 6.05 g Tris, 1.00 g sodium azide, 13.30 g NaCl, 1.00 g bovine serum albumen in 1000 ml distilled water). The chromozym chromogenic substrate (TGPApNA) was diluted to 2 mM with distilled water.

#### Preparation of the blank plasma

Blank plasma was obtained from the orbital sinus of rats that had not been injected with recombinant hirudin, using procedures described by Van Herk et al (1998). 3 ml of the blank plasma sample was treated with 0.3 ml of 1 M HCl at 65°C for 5 min. The blank plasma samples were rapidly cooled to room temperature. After an addition of 0.3 ml of 1 M NaOH, the blank samples were centrifuged at 5000 rpm for 15 minutes. The supernatant was collected and diluted 20-fold with 50 mM Tris-buffer pH 8.3 to serve as the blank samples.

#### Preparation of the sample from rat plasma

The rat plasma sample preparation followed the same procedure as that for the blank, described above, except 0.5 ml of the rat plasma was treated with 0.05 ml of 1 M HCl, and 0.05 ml of 1 M NaOH accordingly.

The rH concentration in plasma was determined as previously described [11].

### 2. ELISA method

#### Solution preparation

Wash buffer was prepared by dissolving the content of the PBS/Tween 20 package according to the manufacturer's instructions. The sample buffer (T-buffer) was prepared by dissolving 3 g of bovine serum albumin in 100 ml of wash buffer.

#### Preparation of the rat plasma blank

Plasma was obtained from rats that have not been injected with recombinant hirudin from the orbital sinus. The plasma blank was diluted 100-fold with saline, followed by an additional 10-fold dilution with T-buffer.

The rH concentration in plasma was determined as previously described [10].

### 3. Total radioisotope assay and trichloroacetic acid pre-treated total radioisotope assay method

#### Standard curve of total radioisotope assay and the trichloroacetic acid pre-treated total radioisotope assay

The ^125^I-recombinant hirudin solutions prepared above were diluted with saline to obtain a solution of specific radioactivity of 725 Bq/ml and recombinant hirudin was added to obtain a standard stock solution concentration of 0.4 g/L. The standard stock solution was diluted to concentrations of 0.026, 0.128, 0.64, 3.2, 16, and 80 μg/ml with diluted rat plasma blank. 100 μl of the standard solution was added to each sample tube. The radioactivity of the samples was measured by a gamma counter. After an addition of 100 μl of trichloroacetic acid solution, the samples were centrifuged at 10,000 rpm for 10 minutes. The supernatant was discarded and the radioactivity of the precipitate was determined.

The plasma samples were analyzed using the total radioisotope assay and the trichloroacetic acid pre-treated total radioisotope assay method. The radioactivity was plotted against the corresponding concentrations of recombinant hirudin standard to generate the standard curve. The recombinant hirudin concentration was calculated using this standard curve.

### 4. Statistical methods

The statistical comparison between two groups was analyzed by the ANOVA variance analysis and the multiple comparisons between groups were analyzed by the LSD method [14].

## Results

### 1. Chromogenic substrate assay method

#### Assay validation

The results displayed a good relationship between absorbance (Y) and concentration (X, ng/ml) with a linear equation of Y  = −0.01X +0.541 (r = 0.99, n = 5). The limit of detection (LOD) was 3.12 ng/ml. At recombinant hirudin concentrations of 40, 12.5 and 3.125 ng/ml, the recombinant hirudin recovery was 100.6±3.04%, 93.9±8.05% and 89.8±32.9%, respectively; the intra-day variation RSDs were 5.01%, 4.27% and 6.33%, respectively; and inter-day variation RSDs were 12.6%, 11.9% and 12.3%, respectively. The diluted recoveries were 90.3±2.77%, 93.7±0.50%, 92.0±1.79%, 95.7±3.94% and 97.8±4.24% of 0, 5, 10, 20 and 40-fold dilution, respectively, of the initial concentrations of 12.5 ng/ml with plasma blank.

#### The pharmacokinetics of recombinant hirudin in plasma

**Figure 1:**
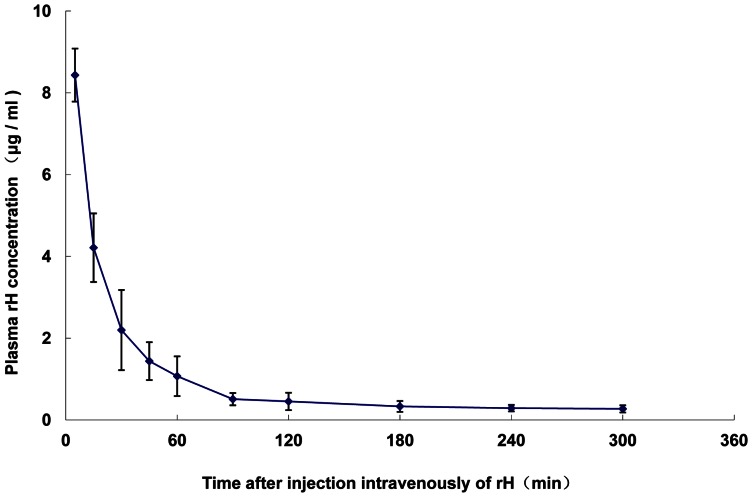
The recombinant hirudin concentration in plasma by CSA. The concentration–time curve of recombinant hirudin in the plasma of rats that had been injected intravenously with 125I-recombinant hirudin, as determined by the chromogenic substrate assay. Each point represents mean±SD (n = 5).

The concentration–time curve (Figure 1) showed that after intravenous administration, the recombinant hirudin in plasma decayed bi-exponentially, with an extremely rapid initial phase followed by a relatively slow elimination phase. A two-compartment model for recombinant hirudin was further confirmed. The main pharmacokinetic parameters are presented in Table 1. The distribution half-life (*t*_1/2_α) was 7.59±4.56 minutes. The elimination half-life (*t*_1/2_β) was 47.0±22.2 minutes. These results suggest that the recombinant hirudin was distributed from the central to the peripheral compartment and eliminated rapidly from the body. These results are similar to those of the chromogenic substrate assay below except that the area under the curve was 204.5±19.9 mg/L/min by the chromogenic substrate assay and 575.2±39.4 mg/L/min by ELISA.

**Table 1:** Pharmacokinetic parameters determined by the chromogenic substrate, ELISA, the total radioisotope assay and the trichloroacetic acid pre-treated total radioisotope assay.

Pharmacokinetic parameters	Chromogenic substrate assay	ELISA	Total radioisotope assay	The trichloroacetic acid
	(n = 5)	(n = 4)	(n = 5)	pre-treated total radioisotope assay (n = 5)
*t*_1/2_α (min)	7.59±4.56	6.41±2.60	3.69±1.71	4.57±1.73
*t*_1/2_β (min)*	47.0±42.2	47.3±13.1	701.9±198.8	724.9±81.2
V_d_ (L/kg)*	0.17±.06	1.24±0.23	0.04±0.02	0.09±0.04
AUC_(0–t)_ (mg/L×min)*	204.5±19.9	575.2±39.4	4189±139.1	2329±95.9

*t*_1/2_α: Half-life of distribution phase; *t*_1/2_β: Half-life of elimination phase; V_d_: Volume of apparent distribution; AUC_(0–t)_: Area under the concentration-time curve from zero to infinite time. Data are expressed as mean ± SD.

* The difference between the four methods is p<0.05.

### 2. ELISA method

#### Assay validation

The absorbance (Y) versus concentration (X, ng/ml) displayed a linear relationship, as described by the equation of Y = −0.63X +0.05 (r = 0.99, n = 5). The limit of detection (LOD) was 0.25 ng/ml. The recombinant hirudin recovery was 102.1±1.61%, 104.9±3.10%, and 86.6±3.05% at the concentrations of 3.0, 1.0 and 0.25 ng/ml, respectively. The intra-day variation RSD were 1.7%, 4.5%, and 4.9%, and inter-day variation RSD were 11.9%, 12.32%, and 12.5% respectively at those concentrations.

#### The recombinant hirudin pharmacokinetic in plasma

**Figure 2:**
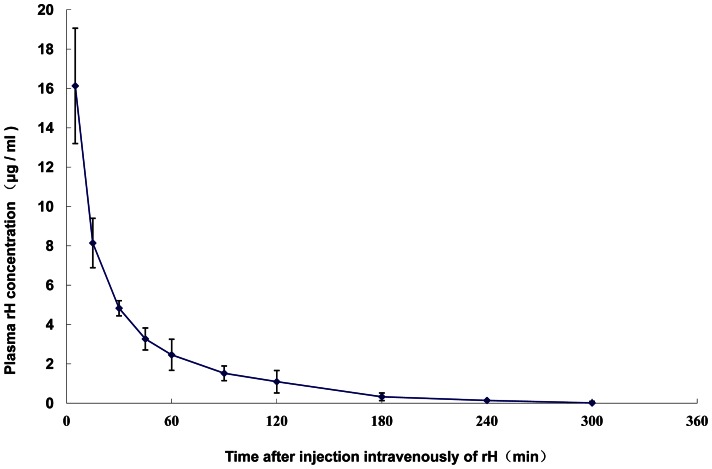
The recombinant hirudin concentration in plasma by ELISA. The concentration–time curve of recombinant hirudin in the plasma of rats that had been injected intravenously with 125I-recombinant hirudin, as determined by ELISA. Each point represents mean±SD (n = 4).

The plasma concentration–time curve (Figure 2) from the ELISA assay showed that, similar to the concentration-time curve derived from the chromogenic substrate assay, the ^125^I-recombinant hirudin decayed bi-exponentially with an extremely rapid initial phase followed by a relatively slow elimination phase. The two-compartment model for recombinant hirudin was further confirmed. The main pharmacokinetic parameters are presented in Table 1. The distribution half-life (*t*_1/2_α) was 6.42±2.60 minutes. The elimination half-life (*t*_1/2_β) was 47.3±13.1 minutes. In agreement with the results obtained from chromogenic assay, these results also suggest that the recombinant hirudin was distributed from central to peripheral compartment, and eliminated rapidly from the body.

### 3. Total radioisotope assay and the trichloroacetic acid pre-treated total radioisotope assay

Radioactivity (Y) versus concentration (X, µg/ml) displayed a linear relationship, as described by the equation of Y = 141.64X −63.93 (r = 1.00, n = 5) from the total radioisotope assay); and Y = 24.15X −6.95 (r = 1.00, n = 5) from the trichloroacetic acid pre-treated total radioisotope assay). The limit of detection (LOD) was 0.026 µg/ml. The intra-day variation RSD were 1.7%, 4.5% and 4.9%, and inter-day variation RSD were all less than 10%. The recombinant hirudin recovery was 98.5±0.5%, as determined by the total radioisotope assay method.

**Figure 3:**
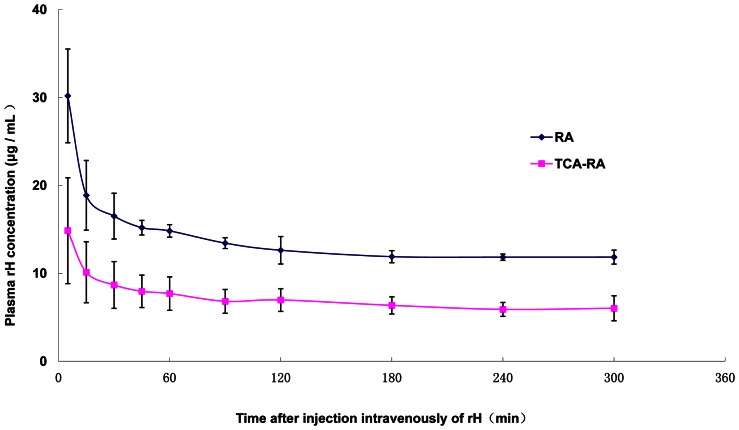
The recombinant hirudin concentration in plasma by RA and TCA-RA. The concentration–time curve of recombinant hirudin in the plasma of rats that had been injected intravenously with 125I-recombinant hirudin, as determined by total radioisotope assay (red) and the trichloroacetic acid pre-treated total radioisotope assay (blue). Each point represents mean±SD (n = 5).

The recombinant hirudin concentration–time curve in rat plasma, determined by total radioisotope assay and the trichloroacetic acid pre-treated total radioisotope assay method are shown in Figure 3. The main pharmacokinetic parameters are shown in Table 1. The distribution half-life (*t*_1/2_α) was 3.69±1.71 minutes by the total radioisotope assay and 4.58±1.73 minutes by the trichloroacetic acid pre-treated total radioisotope assay. The elimination half-life (*t*_1/2_β) was 701.9±198.8 minutes and 724.9±81.2 minutes respectively.

## Discussion

The principle of the chromogenic substrate assay method is that thrombin can hydrolyze the chromogenic substrate TGPApNA to release pNA, which has absorption at 405 nm. The thrombin concentration is in excess relative to the recombinant hirudin in the reaction. The quantity of recombinant hirudin is calculated by the difference between the total thrombin and the residual thrombin. The acid treatment of samples at 65°C deactivates the intrinsic thrombin in plasma prior determining the recombinant hirudin concentration, as described previously [15]. The limit of detection of the chromogenic substrate assay method was 3.12 ng/ml, of the ELISA assay method is 0.25 ng/ml; hence these assays could also be used in the pharmacokinetic study of recombinant hirudin in rats.

The isotope tracer method is the classic method for protein (including peptide) drug pharmacokinetic studies. In this paper, the total radioactivity of ^125^I-recombinant hirudin (total radioisotope assay) and the total radioactivity of ^125^I-recombinant hirudin after precipitating the samples with trichloroacetic acid (the trichloroacetic acid pre-treated total radioisotope assay) were employed to study the pharmacokinetic behavior of recombinant hirudin. The pharmacokinetic parameters (except for AUC) showed little difference, indicating the recombinant hirudin sedimentation rate *in vivo* in this study is consistent to that reported in the literature [9], although the trichloroacetic acid concentration was low (less than 20%). The recombinant hirudin could not be completely precipitated because of its low molecular weight (7000 Da) and higher solubility.

The three main pharmacokinetic parameters calculated by the three methods are listed in Table 1 and the multiple comparisons of the pharmacokinetic parameters within each group are listed in Table 2. The AUC _(0–t)_ values, are significantly different from each other, as measured by chromogenic substrate assay, ELISA, the total radioisotope assay and the trichloroacetic acid pre-treated total radioisotope assay. The half-life of the recombinant hirudin is less than 1 hour, as analyzed by the chromogenic substrate assay and ELISA, but the half-life is more than 10 hours, as analyzed by the total radioisotope assay and the trichloroacetic acid pre-treated total radioisotope assay. This discrepancy may be due to long half-life of radioisotope ^125^I. These results suggest that the total radioisotope assay is not a suitable assay to study for pharmacokinetics of recombinant hirudin.

**Table 2:** Multiple comparisons of the pharmacokinetic parameters within each group.

Pharmacokinetic parameters	Group	group			
		1	2	3	4
*t*_1/2_β (min)	2	–	–	*	*
	3	*	*	–	–
	4	*	*	–	–
V_d_ (L/kg)	2	–	–	*	*
	3	*	*	*	–
	4	*	*	–	–
AUC_(0–t)_ (mg/L*min)	2	*	–	*	*
	3	*	*	–	*
	4	*	*	*	–

* The difference between the two groups is p<0.05.

1. The Chromogenic substrate assay; 2. ELISA; 3. the total radioisotope assay; 4. the trichloroacetic acid pre-treated total radioisotope assay.

The statistical comparison between two groups was analyzed by the ANOVA variance analysis and the multiple comparisons between groups were analyzed by the LSD method.
